# A Flow Cytometric Analysis of Vitreous Inflammatory Cells in Patients with Proliferative Diabetic Retinopathy

**DOI:** 10.1155/2013/251528

**Published:** 2013-10-27

**Authors:** Mojca Urbančič, Veronika Kloboves Prevodnik, Daniel Petrovič, Mojca Globočnik Petrovič

**Affiliations:** ^1^University Medical Centre Ljubljana, Eye Hospital, Grablovičeva 46, 1000 Ljubljana, Slovenia; ^2^Institute of Oncology, Zaloška 2, 1000 Ljubljana, Slovenia; ^3^Institute of Histology and Embryology, Medical Faculty, University Ljubljana, Korytkova 2, 1105 Ljubljana, Slovenia

## Abstract

The purpose of this study was to investigate inflammatory cells in vitreous from patients with proliferative diabetic retinopathy (PDR) using flow cytometric analysis. Twenty-eight patients with PDR requiring vitrectomy because of macular traction or tractional retinal detachment were enrolled in the study (*n* = 28), and 6 patients with macular hole (MH) formed the control group. Samples of vitreous and peripheral venous blood were obtained at the beginning of vitrectomy. T lymphocytes were found in vitreous from patients with PDR, and CD4/CD8 ratio was higher in vitreous (median 4.3) compared to blood (median 1.9; *P* = 0.003). No B lymphocytes were detected in vitreous. The percentage of histiocytes/macrophages was significantly higher in vitreous (median 62.1) in comparison with blood (median 5.5; *P* < 0.0001). No lymphocytes were detected in vitreous of the control group. There were more T lymphocytes in vitreous from patients with active PDR. No association between cells in the vitreous and visual acuity improvement after surgery was found. In conclusion, T lymphocytes are found in vitreous from patients with PDR and reflect the activity of PDR but do not seem to predict visual prognosis. Higher CD4/CD8 ratio in vitreous compared to blood from patients with PDR is consistent with local inflammatory response in PDR.

## 1. Introduction

Diabetic retinopathy (DR) is a late microvascular complication of diabetes mellitus and a leading cause of blindness in the working age population. Typical risk factors of DR include hyperglycemia, hypertension, and hyperlipidemia. These factors have been shown to induce retinal inflammation by a variety of mechanisms [1]. There is now general acceptance that DR is a low-grade chronic inflammation [2].

Inflammation is a nonspecific response to injury. Many molecular mediators and functional changes with immune cell and resident macrophage activation are involved in the inflammatory response. In acute inflammation, inflammatory cells contribute to tissue repair. However, leukocytes' prolonged secretion of inflammatory mediators and toxic oxygen radicals in persisting, chronic inflammation can lead to tissue damage [3, 4]. 

Leukocytes are involved in endothelial cell damage, capillary occlusions, and blood-retinal barrier breakdown in DR. Leukocyte adhesion to the endothelial wall and leukostasis is an early event in the development of DR [5]. Leukocyte adhesion molecules are upregulated in the vessels of the diabetic retina and choroid, and consequently inflammatory cells accumulate in the chorioretinal tissues [3]. Studies on diabetic rats revealed increased leukocyte adhesion associated with vascular damage and increased vascular permeability [6–8]. Increased numbers of accumulated leukocytes have been shown to correlate with the retinal capillary damage in spontaneously diabetic monkeys [9]. Accumulations of polymorphonuclear leukocytes have been observed in the lumen of human microaneurysms [10]. 

Leukocytes enhance the formation of new vessels by releasing angiogenic factors and increasing the activity of matrix metallopeptidase [1]. T lymphocytes have been found in fibrovascular membranes of patients with PDR [4, 11] and correlated well with the severity of retinopathy and visual prognosis [3, 11]. Monocytes/macrophages were found in the neovascular tufts [12, 13]. 

It is well known that inflammatory mediators are increased not only in the retina but also in the vitreous. The vitreous actively participates in the etiopathogenesis of DR by means of accumulating inflammatory molecules. A lot of data regarding pathogenesis of DR was obtained indirectly by studying inflammatory molecules in vitreous samples of patients undergoing vitrectomy [14]. Less is known about inflammatory cells in the vitreous, especially about the role of specific subsets of these cells in the pathogenesis of DR. 

Vitreous is composed mainly of collagen fibers, hyaluronic acid, and hyalocytes. Hyalocytes belong to the monocyte/macrophage lineage and have characteristics of tissue macrophages. By acting as modulators of the intraocular immune system and intraocular inflammation, they play a significant role in maintaining the vitreous transparent and avascular. They have been found to be present in diabetic macular edema and PDR [15]. Normally, there are no leukocytes in the vitreous as this is an immune-privileged site [16, 17]. However, when the blood-retinal barrier is disrupted, like in DR, leukocytes gain access to the vitreous. T lymphocytes have been found in most of the vitreous samples from PDR patients and they were not present in the vitreous samples of nondiabetic patients. Moreover, differences in percentages of T lymphocytes between vitreous and peripheral blood were reported by Cantón and coworkers [18].

In our study, we used flow cytometry to investigate inflammatory cells in the vitreous of diabetic patients. Our purpose was to observe pattern changes in lymphocyte subsets (CD3, CD4, CD8, and CD19) and macrophage (CD14) in the vitreous of patients with PDR in comparison with peripheral blood and in comparison with the vitreous of nondiabetic patients. Additionally, we have searched for the possible association of pattern changes in leukocyte subsets with the activity of DR and visual acuity improvement after vitrectomy.

## 2. Patients and Methods

28 patients with PDR requiring vitrectomy because of macular traction/tractional retinal detachment were considered for the study (14 men and 14 women; age: 63.4 ± 12.9 years). Only patients with no vitreous haemorrhage or with vitreous haemorrhage of more than three months duration were enrolled. Exclusion criteria were vitreous haemorrhage of less than three months, recent retinal photocoagulation (less than six months), previous vitrectomy, and glycated haemoglobin of more than 10 percent. Six patients requiring vitrectomy because of macular hole (MH) were enrolled in the study as the control group (3 men and 3 women; age: 72.5 ± 7.8). Patients were not included in the study if they had any other ocular disease or known systemic inflammatory or hematological disease. Informed consent was obtained from all patients. 

All patients had complete ophthalmological evaluation before surgery with best corrected visual acuity (BCVA) determination (Early Treatment Diabetic Retinopathy Study (ETDRS)), slit lamp examination, fundus examination, intraocular pressure measurement, gonioscopy, and optical coherence tomography (OCT). Based on ophthalmological evaluation (preoperative and intraoperative), activity of the disease was noted. The activity of the disease was defined as being active retinopathy when there were perfused capillaries in neovascular membranes or inactive, quiescent retinopathy when there were nonperfused capillaries in fibrotic membranes [19]. Data regarding the patient's general condition and diabetes control were obtained from the patient and from the patient's general practitioner or diabetologist. Arterial hypertension was defined by a systolic blood pressure of 140 mm Hg or higher and/or diastolic blood pressure of 85 mm Hg or higher or defined as the condition treated with antihypertensive medications. Hyperlipidemia was defined as total cholesterol higher than 5 mmol/L and/or triglycerides higher than 2 mmol/L or defined as condition treated with hypolipemic medications. Patients were followed after the surgery and BCVA after 6 months was compared to preoperative BCVA. Visual acuity improvement was defined as gain of more than 5 ETDRS letters. For statistical analysis ETDRS visual acuity was converted to logarithm of the Minimum Angle of Resolution (logMAR).

Samples of undiluted vitreous and samples of peripheral venous blood were obtained at the beginning of vitrectomy. Undiluted vitreous samples were obtained by aspiration into a syringe attached to a vitreous cutter before the infusion line with balanced salt solution was opened. 0.5 mL of undiluted vitreous sample was then put immediately into 1 mL of cell media (4.5% bovine serum albumin and 0.45% EDTA in phosphate buffer solution with 50 IE/mL of penicillin). For microscopic examination with light microscope two cytospins were prepared (Giemsa and Papanicolaou staining method) and samples with erythrocytes present were considered as blood contaminated. Based on this vitreous sample contamination, patients with PDR were further divided into two groups: vitreous samples of 12 patients were considered as noncontaminated with blood (3 men and 9 women; age: 67.3 ± 14.9); blood contaminated vitreous samples of 16 patients formed the other group (11 men and 5 women; age: 60.4 ± 10.7).

Flow cytometric analysis (FCA) for markers CD45 (leukocyte common antigen), CD14 (monocyte/macrophage marker), CD3 (T lymphocyte marker), CD19 (B lymphocyte marker), kappa (kappa light chain B cell marker), lambda (lambda light chain B lymphocyte marker), CD4 (T helper lymphocyte marker), and CD8 (T cytotoxic lymphocyte marker) was done from vitreous and peripheral venous blood samples. Samples of undiluted vitreous were prepared according to the modified protocol adopted for cytological samples at the Institute of Oncology, Ljubljana, Slovenia [20]. Antibodies by BD Biosciences were applied (Table 1). Peripheral venous blood samples were prepared according to red blood cell lysis protocol for peripheral blood. 2.5 mL of TRIS ammonium chloride solution for erythrocyte lysis was first added to 100 mL of peripheral venous blood. After 10-minute incubation at room temperature in dark place, samples were centrifuged for 5 minutes at 1500 turns per minute. Supernatant was discarded and washed with 2.5 mL of buffer (Cell Wash, BD Biosciences). Antibodies (BD Biosciences, Table 1) were added and samples then incubated in dark at room temperature for 20 minutes. After 20 minutes superfluous antibodies were washed away with 2.5 mL of buffer and 300 *μ*L of buffer (Cell Wash, BD Biosciences) was added. FCA was done with flow cytometer FACSCanto II (Becton Dickinson, San Jose, CA). Measurement results were analyzed with FACSDiva programme.

Measurement data in our study did not meet the normality assumption, so median and range between minimum and maximum variable were used for the description of data. The comparison of related samples was done with Wilcoxon signed rank test. Mann Whitney *U* test was used for assessing differences between independent groups. A *P* value of less than 0.05 was considered statistically significant.

The study was approved by the National Ethical Committee (National Ethical Committee number 118/12/2011) and was performed in compliance with the Helsinki declaration. 

## 3. Results

Clinical data of patients enrolled in the study are presented in Table 2.

Vitreous samples were first evaluated by light microscope. The samples with erythrocytes present under microscopic examination were considered as blood contaminated. According to these findings, patients with PDR were divided into two groups: blood noncontaminated group (nPDR) (*n* = 12) and blood-contaminated group (cPDR) (*n* = 16). In the nPDR group 0–10 lymphocytes and 1–30 hystiocytes were found on microscopic examination. In the control group (MH group) no erythrocytes were found; therefore these samples were considered uncontaminated with blood. No lymphocytes and only 0–2 hystiocytes were found in this group. 

FCA measurement results of vitreous samples and peripheral venous blood samples from patients with PDR are presented in Table 3. Since no kappa and lambda chains were detected in vitreous samples, they are not presented in the tables. 

The paired comparison of vitreous and peripheral venous blood samples from patients with PDR (*n* = 28) showed significant differences in the number of CD45+ cells and in the percentages of CD14+, CD19+, and CD8+ cells between vitreous and blood samples (*P* < 0.0001). Ratio between CD14+ cells and lymphocytes was higher in vitreous. Percentages of lymphocytes, CD3+, and CD4+ cells were not significantly different, but CD3/CD19 ratio and CD4/CD8 ratio were significantly higher in vitreous compared to blood (Table 3). 

The comparison of blood noncontaminated and blood contaminated vitreous samples is presented in Table 4. There were more leukocytes (CD45+ cells) in blood-contaminated samples and further immunophenotyping revealed that this was mainly due to CD3+ cells. CD4/CD8 ratio was not significantly different between both groups.

Only blood noncontaminated vitreous samples were used for further comparison between patients with PDR and nondiabetic patients with MH and for searching for a possible association between cell pattern in the vitreous and the activity of the disease and visual gain after surgery.

The comparison of blood noncontaminated vitreous samples of patients with PDR (nPDR group) and patients with MH (MH group) is presented in Table 5. Significantly more CD45+ cells were found in vitreous samples of patients with PDR with highly variable percentage of lymphocytes present. There were no lymphocytes detected in the vitreous samples of the control MH group, except in two patients where the percentage of lymphocytes was less than 3 and further immunophenotyping did not show CD3, CD19, CD4, or CD8 positive cells. The percentages of CD14+ cells were similar in both groups. Peripheral venous blood samples were also compared and there were no significant differences in the number of CD45+ cells and in the percentages of observed cells between the two groups.

Active neovascularization was observed in 17 patients with PDR (6 noncontaminated with blood and 11 blood contaminated); 11 patients had quiescent PDR (6 noncontaminated and 5 blood contaminated). When comparing blood noncontaminated vitreous samples from patients with active retinopathy and from patients with quiescent retinopathy (presented in (Table 6)), there were more CD45+ cells and the percentages of CD3+, CD4+, and CD8+ cells were significantly higher in vitreous samples from patients with active PDR. The percentages of lymphocytes were very low in samples from patients with quiescent PDR and further immunophenotyping was negative for CD19+ cells in all samples and negative for CD3+ cells in five samples. In the sample from patient with quiescent PDR where CD3+ cells were detected, only CD4+ cells were detected with further immunophenotyping.

Visual acuity improved 6 months after surgery in 23 (82%) patients with PDR (8 patients with blood noncontaminated vitreous samples and 15 patients with blood contaminated vitreous samples), remained the same in 3 (11%) patients (2 patients with noncontaminated vitreous samples and 1 contaminated), and worsened in 2 (7%) patients (both noncontaminated). The average BCVA before surgery was 1.2 ± 0.62 logMAR and 6 months after surgery 0.75 ± 0.55 logMAR. Considering only patients with blood noncontaminated vitreous samples, preoperative average BCVA was 1.02 ± 0.68 and postoperative BCVA six months later was 0.86 ± 0.57. No association was found between cells in the vitreous and visual acuity improvement after surgery. 

## 4. Discussion

In our study, differences in the percentages of lymphocytes and macrophages in the vitreous between patients with PDR and either peripheral blood of patients with PDR or vitreous from nondiabetic patients using flow cytometry were observed. T lymphocytes were found in most vitreous samples from patients with PDR, and CD4/CD8 ratio was higher in vitreous samples in comparison with peripheral blood. To our knowledge, this is the first report of higher CD4/CD8 ratio in vitreous in patients with PDR indicating the importance of local inflammation. In our study, higher percentages of T lymphocytes were associated with active PDR. Similar findings, that is, higher percentages of T lymphocytes (CD4+ and CD28+ cells) in the vitreous in comparison with peripheral blood in patients with PDR, were reported by Cantón and coworkers [18]. In their study, however, all the patients with PDR reported had the quiescent form of PDR [18]. CD4/CD8 ratio was approximately two times higher in vitreous (4.3) compared to blood (1.9) in our study. This suggests that inflammatory cells in vitreous are under the influence of local environment. Considering diabetic retinopathy as low-grade chronic inflammation, these results are in concordance with some studies reported in uveitic patients. T lymphocytes are known to participate in all types of uveitis [21, 22]. Moreover, intraocular inflammation was reported to be mediated by activated CD4+ T cells [21, 22]. CD4/CD8 ratio higher than 4.0 has been reported to have positive predictive value of 70% in patients having noninfectious uveitis [23, 24]. High CD4/CD8 ratio (higher than 3.5) in the vitreous of patients with ocular sarcoidosis has also been found to be of high diagnostic value with sensitivity and specificity of 100% and 96.3%, respectively [25]. 

There were significantly more CD45+ cells (leukocytes) in the vitreous of patients with PDR (nPDR group) compared to nondiabetic patients with MH. There were no lymphocytes detected in the vitreous of nondiabetic patients. Lymphocytes were detected in all vitreous samples from patients with PDR, although there were also five samples with small percentages of detected lymphocytes, and further phenotyping was negative. Nevertheless, these results are in concordance with the concept that blood-retinal barrier breakdown is necessary for inflammatory cells to gain access into the vitreous, since leukocytes have an active role in blood-retinal barrier breakdown during inflammation [5]. 

Great variations in the percentages of lymphocytes in vitreous from patients with PDR (nPDR) (0.4 to 55.5) in our study seem to be in association with the activity of PDR. T lymphocytes were detected in all samples with active PDR and only in one sample with quiescent PDR. Our findings are in contrast with the findings of Cantón and coworkers who reported T lymphocytes in 55% of patients with no vitreous haemorrhage, but all patients in whom T lymphocytes were detected had quiescent PDR [18]. Cantón and coworkers assumed that T lymphocytes infiltrating the vitreous cavity had a protective role in the outcome of PDR since patients in whom T lymphocytes were detected in the vitreous had quiescent disease and better outcome in terms of early bleeding after vitrectomy [18]. Based on our results, we were not able to confirm Cantón's conclusions. In our study, T lymphocytes were associated with active PDR. None of our patients had early postoperative bleeding. No association between cells in the vitreous and visual acuity improvement after surgery was found in our study to support the idea of the protective role of T lymphocytes. Visual acuity depends on many factors, with one of them being the preservation of photoreceptors in macula, which was not evaluated in this study but could be in relation with local inflammation in the retina itself. It has been shown in the study of Kase et al. [11] that high infiltration of fibrovascular membranes with T lymphocytes in patients with PDR is associated with poor visual prognosis. In our opinion, T lymphocytes in vitreous cavity might reflect the activity of PDR but could not be used as a predictor of visual prognosis.

We did not observe significant difference in the cell pattern in blood samples from patients with PDR (nPDR) and nondiabetic patients. Moreover, we did not find statistically significant differences in either CD3/CD19 ratio or CD4/CD8 ratio in patients with PDR. Our findings are consistent with some previous reports [26]. Additional diagnostics is necessary to detect systemic inflammation in type 2 diabetes mellitus. 

Higher percentages of CD14+ cells (macrophage/histiocyte) in vitreous in comparison to blood were observed in patients with PDR. These percentages were similar regardless of blood contamination. Macrophages are known to play an important role in the pathogenesis of proliferative vitreoretinal disorders and they have been found in proliferative membranes in patients with PDR [27]. Increased number of preretinal hyalocytes (vitreous macrophages) in mice exposed to uncontrolled hyperglycemia was reported by Vagaja and coworkers [28]. Histiocytes (macrophages) and T lymphocytes were found to be a major sign of intravitreal inflammation in various conditions presenting as vitreous opacity [29]. However, we did not observe higher percentages of CD14+ cells in vitreous from patients with PDR in comparison with vitreous from nondiabetic patients, which leads us to the conclusion that inflammation in diabetic patients does not have a significant effect on the number of CD14+ cells in vitreous.

To conclude, differences in the percentages of lymphocytes and macrophages in the vitreous between patients with PDR and either peripheral blood of patients with PDR or vitreous from nondiabetic patients using flow cytometry were observed. T lymphocytes were found in the vitreous of patients with PDR, reflecting the activity of PDR, but they do not seem to be a predictor of visual prognosis. Higher CD4/CD8 ratio in vitreous compared to blood confirms the hypothesis that local intravitreal inflammation plays an important role in the development of PDR.

## Figures and Tables

**Table 1:** Antibodies for 8-colour immunophenotyping of vitreous and peripheral venous blood samples.

Test tube	FITC	PE	PerCP-Cy5.5	APC	PE-Cy7	APC-Cy7
	mAbs	mAbs	mAbs	mAbs	mAbs	mAbs
1	Kappa* + CD4*	Lambda* + CD8*	CD45*	CD14**	CD19**	CD3**

Legend: mAbs: monoclonal antibodies, FITC: fluorescein isothiocyanate, PE: phycoerythrin, PerCP-Cy5.5: peridinin chlorophyll protein-Cy5.5, APC: allophycocyanin, PE-Cy7: phycoerythrin-cyanin 7, APC-Cy7: allophycocyanin-cyanin 7; *5 *μ*L monoclonal antibody was added; **3 *μ*L monoclonal antibody was added.

**Table 2:** Data of patients.

	PDR (*n* = 28)	MH (*n* = 6)
Age (years)	63.4 ± 14.9	72.5 ± 7.81
Gender	14 men (50%), 14 women (50%)	3 men (50%), 3 women (50%)
Duration of diabetes (years)	15.8 ± 8.8	0
HbA1c (%)	7.8 ± 1.0	0
BMI (kg/m^2^)	30.0 ± 4.9	25.0 ± 5.2
Incidence of arterial hypertension	26 (92.8%)	4 (66.7%)
Incidence of hyperlipidemia	14 (50%)	2 (33.3%)
Incidence of insulin therapy	20 (71.4%)	0

Legend: PDR: patients with proliferative diabetic retinopathy; MH: patients with macular hole; HbA1c: glycated haemoglobin; BMI: body mass index.

**Table 3:** Paired comparison of vitreous and blood samples in patients with PDR (median, minimum–maximum; Wilcoxon signed rank test).

	Patients with PDR (*n* = 28)		
	Vitreous	Blood	*P* value
CD45+ (number)	648.0 (181.0–4926.0)	106869.5 (85539.0–265227.0)	**0.0001**
Ly (%)	9.9 (0.4–89.6)	26.8 (8.5–82.1)	0.158
CD14+ (%)	62.1 (5.3–87.7)	5.5 (1.2–12.0)	**0.0001**
CD19+ (%)	0 (0–9.7)	8.9 (2.5–83.0)	**0.0001**
CD3+ (%)	82.4 (0–100.0)	71.0 (12.6–95.6)	0.362
CD3/CD19	83.5 (14.1–100.0)	8.2 (3.1–27.0)	**0.0001**
CD4+ (%)	75.9 (0–100.0)	62.4 (24.7–78.4)	0.452
CD8+ (%)	14.0 (0–66.7)	33.1 (16.9–68.2)	**0.0001**
CD4/CD8	4.3 (0.5–100.0)	1.9 (0.4–23.0)	**0.003**

Legend: PDR: proliferative diabetic retinopathy; CD45+: leukocytes; Ly: lymphocytes; CD14+: macrophages; CD19+: B lymphocytes; CD3+: T lymphocytes; CD4+: T helper lymphocytes; CD8+: T cytotoxic lymphocytes.

**Table 4:** Comparison of blood non-contaminated (nPDR) and contaminated (cPDR) vitreous samples of patients with PDR (median, minimum–maximum; Mann Whitney *U* test).

	Vitreous		
	nPDR (*n* = 12)	cPDR (*n* = 16)	*P* value
CD45 (*n*)	401.0 (181.0–1644.0)	938.0 (273.0–4926.0)	**0.029**
Ly (%)	3.3 (0.4–55.5)	14.7 (0.6–89.6)	**0.048**
CD14all (%)	64.1 (34.0–79.3)	60.5 (5.3–87.7)	0.546
CD19 (%)	0 (0-0)	0 (0–9.7)	0.211
CD3 (%)	57.5 (0–100.0)	88 (0–100.0)	**0.012**
CD3/CD19	84.0 (47.7–100.0)	86.9 (14.1–100.0)	0.723
CD4 (%)	64.0 (0–100.0)	77.5 (0–100.0)	0.065
CD8 (%)	0 (0–26.0)	15.9 (0–66.7)	0.098
CD4/CD8	3.9 (2.2–100.0)	4.6 (0.5–100.0)	0.724

Legend: nPDR: PDR patients with blood non-contaminated vitreous; cPDR: PDR patients with blood contaminated vitreous; CD45+: leukocytes; Ly: lymphocytes; CD14+: macrophages; CD19+: B lymphocytes; CD3+: T lymphocytes; CD4+: T helper lymphocytes; CD8+: T cytotoxic lymphocytes.

**Table 5:** Comparison of samples from the nPDR group and the MH group (median, minimum–maximum; Mann Whitney *U* test).

	Vitreous			Blood		
	nPDR (*n* = 12)	MH (*n* = 6)	*P* value	nPDR (*n* = 12)	MH (*n* = 6)	*P* value
CD45 (number)	401.0 (181.0–1644.0)	132.0 (80.0–429.0)	**0.005**	107079.5 (100911.0–168812.0)	109862.5 (100618.0–127659.0)	0.64
Ly (%)	3.3 (0.4–55.5)	1.0 (0–2.6)	**0.044**	30.2 (10.4–82.1)	23.0 (14.3–63.9)	0.512
CD14all (%)	64.1 (34.0–79.3)	52.75 (17.9–83.0)	0.223	6.0 (2.2–12.0)	8.0 (5.0–21.0)	0.144
CD19 (%)	0 (0-0)	0 (0-0)	0.48	8.4 (3.0–83.0)	8.45 (4.9–19.4)	0.925
CD3 (%)	57.5 (0–100.0)	0 (0-0)	**0.025**	59.9 (12.6–95.6)	61.1 (36.6–67.0)	0.453
CD3/CD19	84.0 (47.7–100.0)			7.5 (3.1–27.0)	4.6 (2.8–13.3)	0.454
CD4 (%)	64.0 (0–100.0)	0 (0-0)	**0.025**	61.3 (24.7–78.4)	58.8 (32.0–75.3)	0.851
CD8 (%)	0 (0–26.0)	0 (0-0)	0.075	32.3 (16.9–68.2)	33.4 (21.4–59.4)	1.0
CD4/CD8	3.9 (2.2–100.0)			2.05 (0.4–8.2)	1.8 (1.3–3.5)	0.963

Legend: nPDR: PDR patients with blood non-contaminated vitreous; MH: patients with macular hole; CD45+: leukocytes; Ly: lymphocytes; CD14+: macrophages; CD19+: B lymphocytes; CD3+: T lymphocytes; CD4+: T helper lymphocytes; CD8+: T cytotoxic lymphocytes.

**Table 6:** Comparison of vitreous samples from nPDR group with active and quiescent PDR (median, minimum–maximum; Mann Whitney *U* test).

	nPDR (*N* = 12)		
	Active PDR (*n* = 6)	Quiescent PDR (*n* = 6)	*P* value
CD45 (*n*)	666.0 (342.0–1644.0)	240.5 (181.0–605.0)	0.055
Ly (%)	17.0 (2.0–55.5)	1.7 (0.4–9.7)	**0.025**
CD14all (%)	48.8 (34.0–77.0)	66.2 (53.4–79.3)	0.2
CD19 (%)	0 (0–1.0)	0 (0-0)	0.317
CD3 (%)	82.5 (68.0–100.0)	0 (0–47.4)	**0.003**
CD3/CD19	88.5 (68.0–100.0)		
CD4 (%)	73.9 (57.0–100.0)	0 (0–100.0)	**0.037**
CD8 (%)	20.0 (0–26.0)	0 (0-0)	**0.007**
CD4/CD8	3.85 (2.2–100.0)		

Legend: nPDR: PDR patients with blood non-contaminated vitreous; CD45+: leukocytes; Ly: lymphocytes; CD14+: macrophages; CD19+: B lymphocytes; CD3+: T lymphocytes; CD4+: T helper lymphocytes; CD8+: T cytotoxic lymphocytes.
